# Combined surgical and medical treatment in an adolescent with severe gynecomastia due to excessive estradiol secretion: a case report

**DOI:** 10.1186/s12887-019-1887-7

**Published:** 2019-12-26

**Authors:** Jung-Eun Moon, Cheol Woo Ko, Jung Dug Yang, Joon Seok Lee

**Affiliations:** 1 Department of Pediatrics, School of Medicine, Kyungpook National University, Kyungpook National University Hospital, Daegu, Republic of Korea; 2 Department of Plastic and Reconstructive Surgery, Kyungpook National University, School of Medicine, 130 Dongdeok-ro, Jung-gu, Daegu, 41944 Republic of Korea

**Keywords:** Pediatric gynecomastia, Excessive estradiol, Severe gynecomastia

## Abstract

**Background:**

Gynecomastia develops due to the reversed estradiol-to-Testosterone ratio in adolescence, and symptoms typically improve within 2 years. The causes vary widely, including estrogen excess and tumors, and surgical treatment is usually given in late adolescence because postoperative symptoms may recur in adolescents. This study reports a case of a pediatric patient with severe gynecomastia due to excessive estradiol secretion who showed a positive outcome after receiving surgical treatment combined with aromatase inhibitor administration.

**Case presentation:**

A 9-year old boy visited to the Department of Pediatric Endocrinology for breast budding. At that time, the patient showed breasts at Tanner stage II and no abnormality on hormone tests. During a follow-up, both gynecomastia had progressed to Tanner stage III–IV at age 13. Tamoxifen 10 mg bid was administered; however, the condition rapidly progressed to Tanner stage V at 13.5 years. The evaluation of pathologic gynecomastia showed an increase of estradiol to 296 pg/mL with normal range 10 ~ 36 pg/mL and microlithiasis in both testes. As the condition worsened, total mastectomy was performed at the age of 13.5 years. Based on the assessment that elevated aromatase activity had induced breast budding, we changed the medication to anastrozole (Arimidex) 1 mg once a day, after which the estradiol level improved to 38.5 pg/mL and was maintained well in the two-year postoperative follow-up.

**Conclusions:**

This case report shows a combined plastic surgery and appropriate medical management bring a positive outcome in severe gynecomastia patient.

## Background

Gynecomastia, enlargement of the breast tissue in men, is often found in infancy, adolescence, and late adulthood, with 50–60% of incidences occurring in adolescence [1–3]. Gynecomastia is caused by ductal epithelial hyperplasia and increased growth of stromal and periductal connective tissue due to elevated estradiol activity and lowered androgen activity [1, 4]. The etiology has not been clearly identified, but it is predicted to be from abnormal testosterone-to estrogen ratio and it is believed to be related to increased peripheral conversion of testosterone to estradiol as testosterone levels rise during puberty [5]. Although gynecomastia is a physiologic finding that spontaneously resolves in most cases [4, 6], it is important to rule out pathologic gynecomastia in severe and progressive cases. Causes of gynecomastia vary and include hypogonadism, thyroid disease, hyperprolactinemia, estrogen excess, and tumors [7]. Estrogen biosynthesis involves a conversion to steroid precursor androgens and the conversion of androgens to estrogens by enzymatic aromatase activity [8]. The increase in excess estradiol also results from elevated aromatase activity, and its causes include the elevated secretion of enzymes in Sertoli and Leydig cell tumors and aromatase gene mutation [9, 10]. Pathologic gynecomastia is uncommon, and it is crucial to perform appropriate tests to diagnose and provide proper treatment. For idiopathic gynecomatia patients in adolescent period, the first line choice is Tamoxifen, and in aromatase excess patients, aromatase inhibitor is used and if there is a tumor, then the tumor is removed by principle. However, the decision of the best therapeutic approach should be made considering the psychological distress in patients with gynecomastia [11]. In cases where patients experience psychosocial distress and trauma, surgical treatment can be performed, but it should be done in late adolescence after other causes have been ruled out.

There have been many reports on surgical methods for breasts, including various plastic surgery techniques performed to enhance the size, shape, or esthetics of the breasts, and the commonly reported oncoplastic breast reconstruction technique, which is often performed in breast cancer patients. In cases of mild or focal gynecomastia, which are common, liposuction or subcutaneous shaving should be used, whereas in cases of moderate-to-severe gynecomastia, surgery should be performed by either excising the parenchyma inside the breast while removing the excess skin using an inverted T incision and applying reduction mammoplasty, which is generally used in breast reduction surgeries, or excising to minimize scarring with a peri-areolar incision approach; then, the breast tissue must be biopsied to rule out cancer.

Herein, we report a case of a pediatric patient with severe gynecomastia due to excessive estradiol secretion whose condition resolved by a combination of surgical treatment and aromatase inhibitor. We summarize the clinical and laboratory results from the period between his first visit at 9 years of age and his last postoperative follow-up at 15 years of age and discuss the postoperative course of outcomes.

## Case presentation

**Fig. 1:**
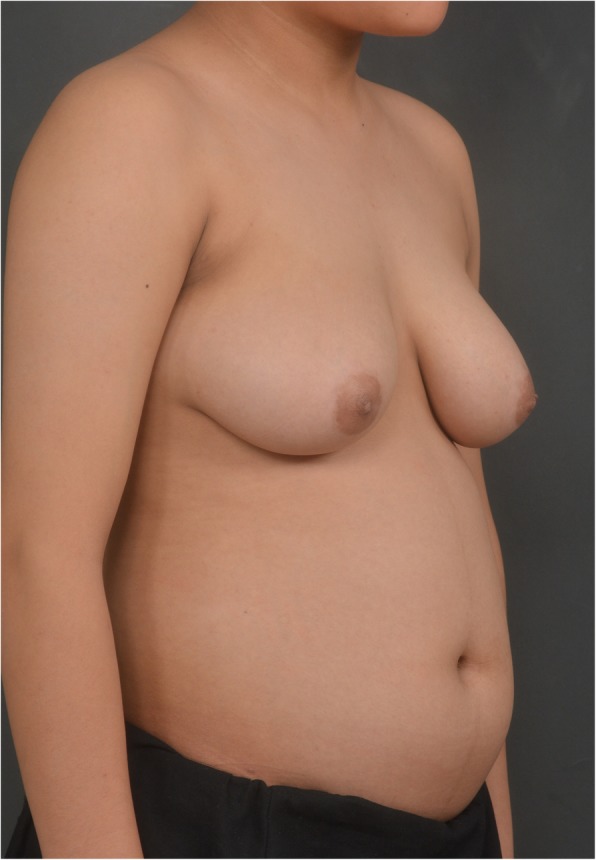
A preoperative photo of pediatric patient with severe gynecomastia (Oblique view)

**Fig. 2:**
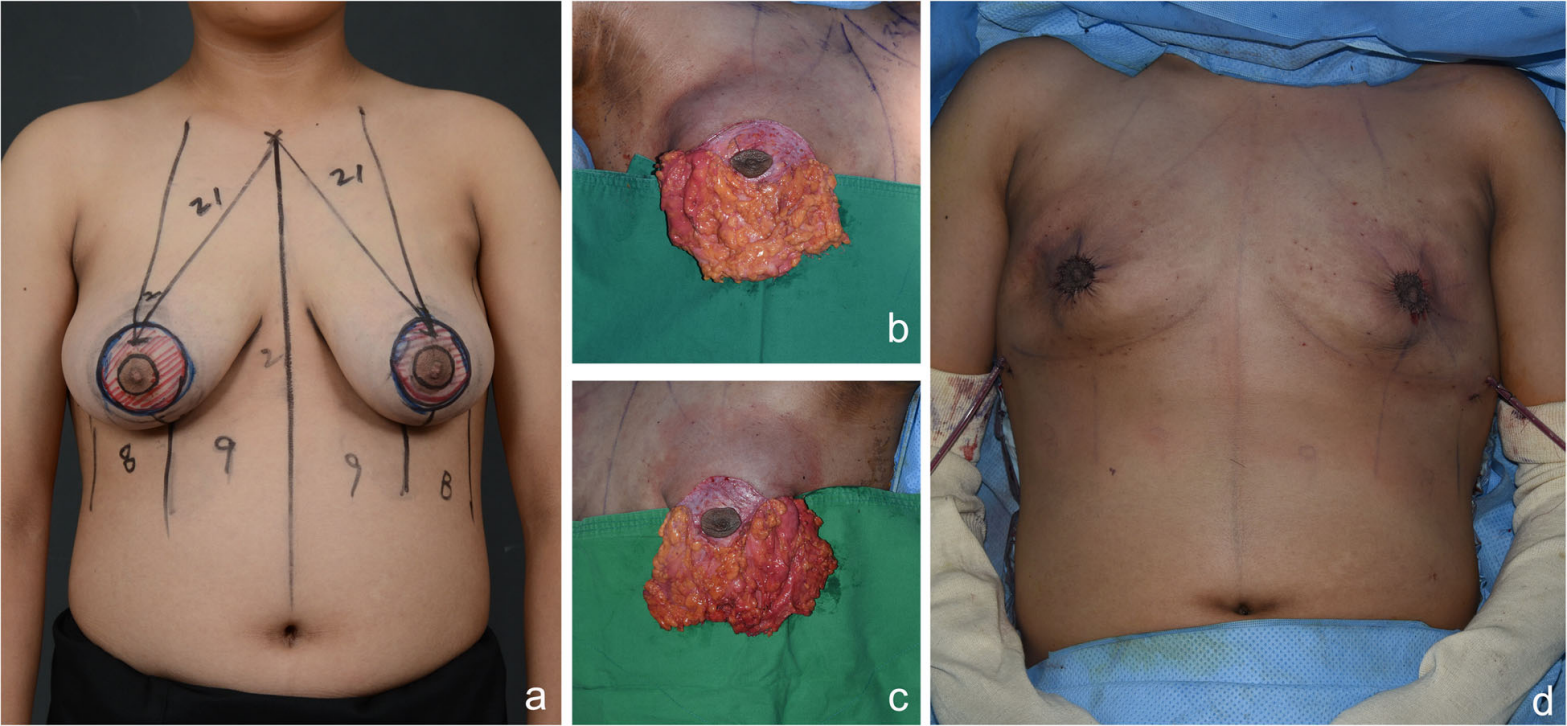
Operative design and postoperative photos of the patient with severe gynecomastia. **a** Preoperative design photo. The red-hatched area was de-epithelized to preserve the pedicle toward the nipple areolar area. **b**, **c** Right and left breasts. **d** Immediate postoperative photo. Purse-string suture was used to reduce the volume of the breast skin flap to a circular shape

**Fig. 3:**
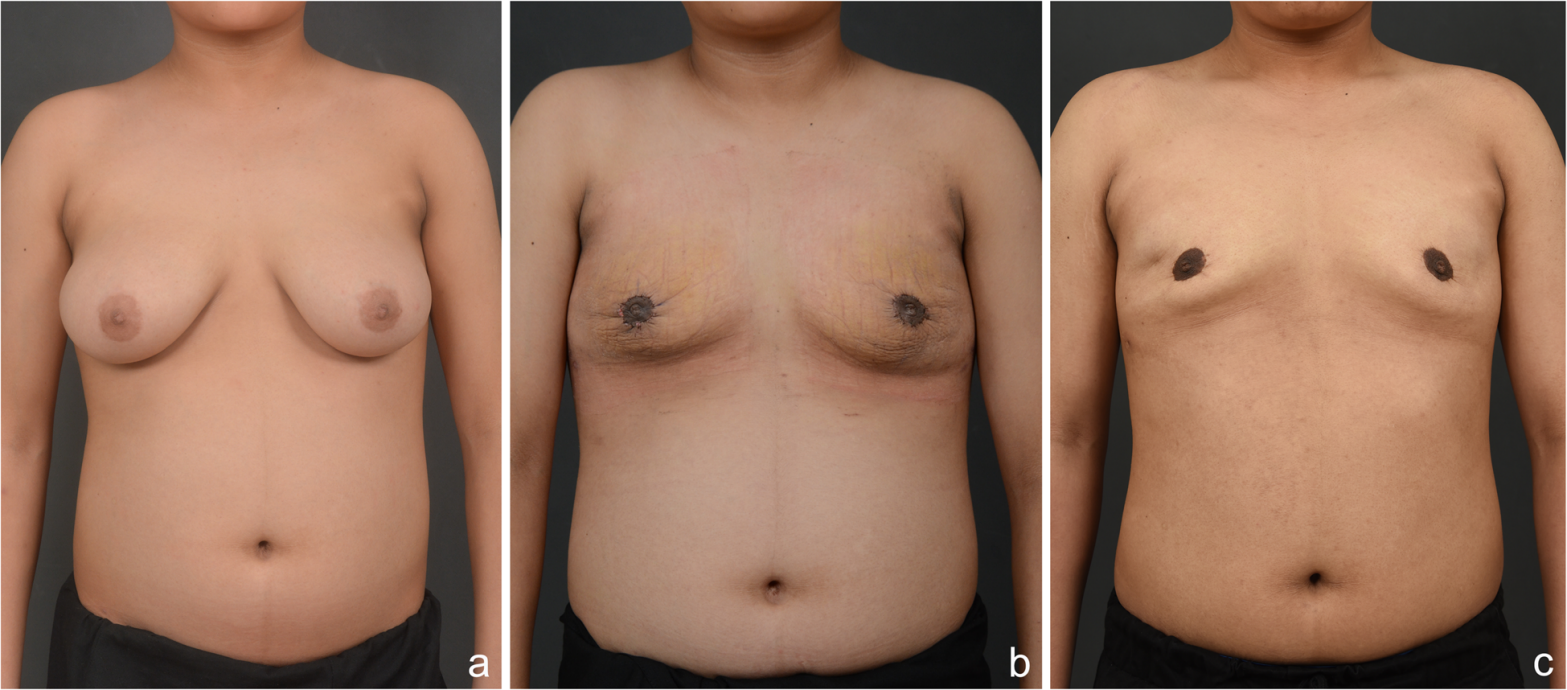
Follow-up photos of pediatric patient with severe gynecomastia. **a** Preoperative finding. **b** Two-week postoperative finding. **c** Two-year postoperative finding

The patient who first visited Kyungpook National University Chilgok-Hospital’s Department of Pediatric Endocrinology at 9 years and 3 months of age, at which time findings showed breasts at Tanner stage II, both testes with 4 mL volume, and no ambiguous genitals. The patient presented with height 144.8 cm (97%), weight 38.4 kg (85%), body mass index (BMI) Z score 0.93. Peripheral chromosome was 46, XY, indicating normal male, and his bone age was 12 years of age according to the Greulich and Pyle atlas. The Leutinizing Hormone Releasing Hormone (LHRH) stimulation test confirmed the prepubertal response. Serum testosterone was < 20 ng/dL with normal range 18 ~ 150 ng/dL, and estradiol was 16 pg/mL with normal range 5 ~ 16 pg/mL. Prolactin, 17-OHP(17-Hydroxyprogesterone), Dehydroepiandrosterone sulfate (DHEA-s) and thyroid test were within normal ranges. During the patient’s six-monthly follow-up visits, the condition rapidly progressed to Tanner stage III–IV at age 13 with estradiol at 60.22 pg/mL with normal range 10 ~ 36 pg/mL and testosterone at 51 ng/dL with normal range 200 ~ 620 ng/dL. As the patient was severely distressed about the symptoms and had difficulty in everyday life, we started treatment with tamoxifen 10 mg bid; the dose was then increased to tamoxifen 20 mg bid after 2 months due to the continued elevation in estradiol and lack of resolution of gynecomastia and his progress was monitored. At 13.5 years of age, the patient was admitted to hospital to check for underlying pathologic disease, as gynecomastia progressed to breast Tanner stage V. He had no family history of gynecomastia or breast hypertrophy, and he was not taking any medications or hormones besides tamoxifen. Abdominal sonography showed no hyperplasia or tumor in the adrenal gland or abnormality in the liver, and testicular sonography showed microlithiasis in both testes but no tumor. Breast sonography also showed normal breast tissue in both breasts with no unusual findings. The laboratory test results included basal LH at 5.17 IU/L with normal range 0.4 ~ 0.7 IU/L, FSH at 6.6 IU/L with normal range 2.6 ~ 11.1 IU/L, estradiol at 296 pg/mL with normal range 10 ~ 36 pg/mL and testosterone at 564 ng/dL with normal range 200 ~ 620 ng/dL. Suspecting aromatase excess syndrome and androgen insensitivity syndrome, we performed target exon sequencing for the *CYP19A1* gene and *AR* gene. There was not found mutation in the *CYP19A1* gene or *AR* gene. At this time, the patient was experiencing severe psychosocial stress about his condition; thus, he was referred to Department of Plastic and Reconstructive Surgery for collaboration in examination, and we set up a surgical plan. First, we performed total mastectomy to excise the breast tissue via the peri-areolar approach to minimize postoperative scar formation considering the patient’s young age and the wishes of both the patient and his guardian who wanted surgical breast reduction to alleviate the stress caused by gynecomastia that was interfering with his everyday life (Fig. 1). We also made a circular incision along the areola periphery while preserving the superomedial pedicle, the most common pedicle of the breast, and the upper halves of the breasts (Fig. 2). The skin flap irregularity after surgery included swelling and contusion; however, it resolved with normal scar formation and contracted as the wound healed. The patient and his guardian reported high satisfaction in a long-term follow-up 2 years after the surgery (Fig. 3).

For the 2 months between the surgery and the time at which the results of the genetic test became available, the patient was on tamoxifen 20 mg bid. Two months after the operation when the patient revisited the hospital, he complained that the operated breasts seemed to be growing again. As the serum estradiol and testosterone levels were 535 pg/mL with normal range 10 ~ 36 pg/mL and 824 ng/dL with normal range 200 ~ 620 ng/dL, respectively.

**Fig. 4:**
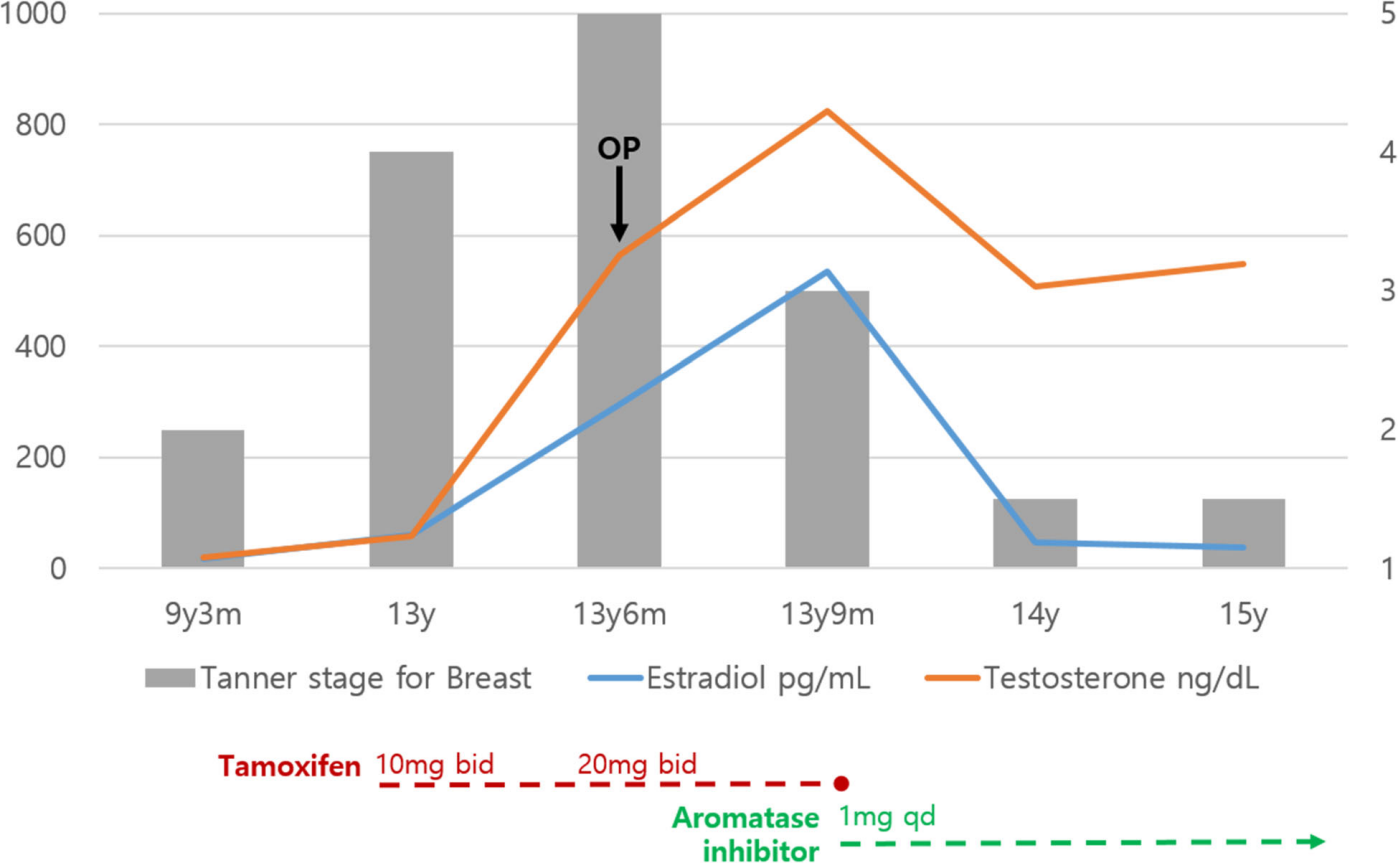
Changes in the serum estradiol and testosterone levels and the breast Tanner stage at each age in the pediatric patient. The figure shows the changes in estradiol and testosterone levels from 9 years and 3 months of age to 15 years of age, including a drastic reduction in the estradiol level after changing tamoxifen with aromatase inhibitor at 13 years and 9 months of age. Following the operation at 13.5 years of age, the Tanner stage of the breast was improved from 5 to 3, followed by a decrease in estradiol at 14 and 15 years of age with symptomatic improvement and maintenance at Tanner stage I–II

Although no mutation was identified in the *CYP19A1* gene exome sequence, it is still possible that there is a mutation in a non-coding region of the *CYP19A1* gene or a related gene. Considering the extremely high level of estradiol and the absence of a tumor, a presumptive diagnosis of elevated aromatase enzyme activity was made, and a change was made from tamoxifen to anastrozole. Follow-up after 3 months showed no progression in the condition with serum estradiol at 46.1 pg/mL with normal range 10 ~ 36 pg/mL, and testosterone at 509 ng/dL with normal range 200 ~ 620 ng/dL. The follow-up visits at one and 2 years after the operation still showed no progression in the breasts, with estradiol at 38.5 pg/mL and testosterone at 548 ng/dL (Fig. 4). Regular urology follow-ups on microlithiasis on both testes have been conducted using sonography, and they have not shown any tumor to date.

The patient was discharged from hospital 3 days after the surgery, and the results of tests performed during the operation showed that the breast tissue had no malignancy. Both the patient and his guardian were highly satisfied with the postoperative shape and minimized scars. The irregularities in the skin flap that were observed immediately after the operation had flattened due to physiologic contracture during the wound healing process. There has been no recurrence or complication to date in the second year after the operation.

## Discussion

In this study, we reported a case of severe gynecomastia in a pediatric patient in whom surgical and endocrine treatments yielded very good outcomes.

The incidence of gynecomastia is fairly high among pediatric patients, most of which have normal physiologic gynecomastia. According to Malhotra’s report, endocrine blood tests can only identify 1.7% of gynecomastia cases as pathologic gynecomastia in pediatric patients, thus offering an unfavorably high cost-to-outcome ratio and making it difficult to justify the need for endocrine screening for pediatric patients who need surgical treatment [12]. However, Einav-Bachar et al. reported that two of 29 pediatric patients with gynecomastia were diagnosed with hyperaromatase syndrome [13]. Given the overall low incidence of hyperaromatase syndrome, this diagnosis rate was high, and the authors suggested that the requisite tests be performed to make the best-informed care decision for gynecomastia [13]. Lazala et al. suggested performing testosterone, estradiol, and gonadotropin tests in pediatric patients presenting with gynecomastia and recommended performing karyotyping if the patient’s testicular volume was < 6 mL [14]. Another study suggested considering a pathologic condition in the case of rapidly growing gynecomastia with breast tissue ≥4 cm in diameter, but there is no guiding recommendation in the literature [15, 16]. Therefore, at the first visit of the patient presented in this study, we checked serum testosterone, serum estradiol, basal LH, and testicular size to identify the cause of pathologic gynecomastia, but the tests yielded no abnormal findings. Based on the guidelines available for gynecomastia evaluation, the patient’s condition was considered physiologic gynecomastia. However, during the course of regular follow-up, his estradiol level was continued to increase during the progression of puberty, and the breast size had reached Tanner stage V in the fourth year of follow-up, making his daily life difficult. Therefore, we performed surgical treatment while making a decision on proper treatment for hormonal abnormality. The patient was also screened for various diseases that were potentially responsible for the excessive estradiol secretion, but no abnormality was found. After taking the aromatase inhibitor, the patient showed lowered estradiol, suggesting that hyperactivity and excess of aromatase was the cause of his condition. The patient was regularly followed up with testis Sonography to observe testicular microlithiasis, as Furness et al. and Tuhan et al. reported that patients with microlithiasis and gynecomastia are at a high risk of testicular tumor [17, 18].

Nuzzi et al. recommended providing information about surgical treatment for patients with gynecomastia progression of 2 years or longer if they experience severe psychosocial distress [19]. Since surgical treatments are not generally considered first-line treatment for pediatric patients, recommending this for our patient was challenging. However, as the patient wanted surgical treatment due to severe psychosocial distress, we planned to combine surgical treatment with hormone therapy. Taking a surgical approach with a pediatric patient allows dramatic reduction of breasts, but most cases of gynecomastia resolve spontaneously, and pathologic gynecomastia can be treated by addressing the underlying causes; therefore, treatment can involve surgically excising the excess breast tissue initially without addressing the endocrinological aspects while obtaining histopathologic findings to make a final diagnosis. A wide range of plastic surgery methods have been reported in relation to gynecomastia. Plastic surgeons can help patients by tailoring the surgery for individual patients due to there being extensive research on cosmetic surgery for breasts and oncoplastic surgery for breast cancer patients. The most commonly reported and available surgery is liposuction; techniques that involve this can minimize scarring and reduce recovery time [20], but it can only be used in mild and localized gynecomastia. In patients with hypertrophic breast tissue resembling women’s breasts, only the reduction of both the skin flap and breast tissue using surgical excision can yield satisfactory outcomes. For women with breast hypertrophy, a design based on the breast reduction technique is generally used. The most common techniques include inverted T incision and vertical incision techniques; although these are very useful for reducing the breast size, they are cosmetically inadequate when applied to severe gynecomastia patients, as they leave long scars. In our case, we excised most of the breast tissue by elevating all breast tissue sagging downward into the mastectomy flap using a peri-areolar incision and reduced the remnant skin by applying the purse-string suture technique to the peri-areolar area to maximize the patient’s satisfaction. The peri-areolar incision left only minor scars, and the remnant skin flap from the mastectomy became less visible as adhesion and contracture proceeded with the healing (Figs. 2, 3). Biopsy of the breast tissue ruled out malignancy. Kasielska-Trojan A. et al. reported that gynecomastia patient showed that life quality improved significantly after the surgery [21]. In our case, patient showed improved psychosocial distress; However, there were limitations such as not being able to compare scoring before and after the surgery through quality-of-life evaluation instrument and the active consideration were not provided to psychological aspects and psychological treatment of the patient during the process of gynecomastia. It reveals that from now on, patients with severe gynecomastia should not only receive endocrine examination and surgical testing and treatment as psychological management also has to be carried out.

This combined treatment of pediatric endocrine therapy and plastic surgery allowed both high patient satisfaction with good cosmetic outcomes and estradiol regulation through medical therapy. The patient is currently maintaining normal hormone levels.

## Conclusions

We report a case of severe gynecomastia in which identification of aromatase excess syndrome leading to pathologic gynecomastia and a combination of endocrine therapy and proactive surgical treatment resulted in positive outcomes in terms of both symptoms and prognosis in a pediatric patient. This case report shows a combined plastic surgery and appropriate medical management bring a positive outcome in severe gynecomastia patient, and it suggests a need for endocrine evaluation in pediatric patients with severe and progressive gynecomastia.

## Data Availability

Data from this study that do not pertain to identifiable patient information are freely available and provided as supplemental material and/or can be obtained by contacting the corresponding author.

## Abbreviations

17-OHP: 17-Hydroxyprogesterone
BMI: Body mass index
DHEA-s: Dehydroepiandrosterone sulfate
LHRH: Leutinizing Hormone Releasing Hormone
T3: Triiodothyronine
T4: Thyroxine
TSH: Thyroid-stimulating hormone
